# Health Literacy Impairment and Awareness of Clinical Pharmacist Services Among Geriatric Tertiary-Care Outpatients: A Cross-Sectional Study

**DOI:** 10.3390/healthcare14131859

**Published:** 2026-06-25

**Authors:** Rajalakshimi Vasudevan, Aziza Alshahrani, Praveen Devanandan, Geetha Kandasamy, Suha S. Alqahtani, Hajar E. Alobaid, Hind M. Alsurraya, Maram S. Alshahrani, Rihanna J. Alshahrani, Amani A. Alwaymani, Lena K. Alghamdi

**Affiliations:** 1 Department of Pharmacology, College of Pharmacy, King Khalid University, Abha 61421, Saudi Arabia; 2 Department of Pharmacy Practice, St. Peter’s Institute of Pharmaceutical Sciences, Hanamkonda 506001, Telangana, India; 3 Department of Clinical Pharmacy, College of Pharmacy, King Khalid University, Abha 61421, Saudi Arabia

**Keywords:** health literacy, geriatric patients, clinical pharmacist services, medication self-management, polypharmacy, patient awareness

## Abstract

**Background:** Health literacy plays an important role in medication understanding, self-management, and engagement with healthcare services among older adults. Limited health literacy may contribute to medication-related problems and reduced utilization of pharmacist-led services in geriatric populations. **Methods**: A cross-sectional, questionnaire-based survey was conducted among geriatric outpatients (≥60 years) attending a tertiary-care teaching hospital in Saudi Arabia. Health literacy was assessed using a four-domain functional tool—covering prescription label comprehension, understanding of healthcare instructions, confidence in completing medical forms, and comprehension of written health information—developed in alignment with established health literacy frameworks, including the Health Literacy Survey—European Union (HLS-EU) model and Baker’s conceptual framework. Participants were classified as having higher health literacy (0–2 domains impaired) or lower health literacy (3–4 domains impaired). Sociodemographic characteristics, clinical burden, medication self-management behaviors, and awareness of clinical pharmacist services were recorded. Multivariable logistic regression was used to identify factors independently associated with lower health literacy. **Results**: A total of 200 participants were included. Impairment in three or more domains was observed in 55.5% of participants. Lower health literacy was independently associated with older age, lower educational attainment, lower income, female sex, multimorbidity, and polypharmacy. Participants with lower health literacy reported higher rates of missed or incorrect medication dosing and unreported adverse drug reactions and lower use of medication management aids. Awareness of clinical pharmacist services and prior exposure to pharmacist counseling were significantly lower among participants with lower health literacy. Willingness to receive pharmacist counseling was higher among participants with higher health literacy and greater awareness of pharmacist roles. **Conclusions**: Health-literacy impairment is common among geriatric outpatients and is associated with medication self-management behaviors and engagement with pharmacist-led services. These findings highlight the relevance of functional health literacy in geriatric medication use and support further research on literacy-sensitive pharmacist-led interventions.

## 1. Introduction

Health literacy is widely recognized as a crucial determinant of health outcomes, especially among older adults who frequently manage multiple comorbidities and complex medication regimens. Limited health literacy impairs an individual’s ability to understand prescription labels, recognize medication purpose, interpret clinical instructions, and make informed decisions regarding their healthcare [1]. Globally, inadequate health literacy in geriatric populations has been linked to poorer adherence, higher rates of medication errors, increased emergency visits, and overall worse health outcomes [2]. The World Health Organization identifies low health literacy as a major barrier to effective chronic disease management and successful patient–provider communication in aging populations [3].

Age-related factors such as declining cognition, reduced vision, multimorbidity, and polypharmacy significantly increase the vulnerability of older adults to medication mismanagement [4]. Studies indicate that nearly 60% of elderly patients struggle with understanding basic dosing instructions, and over one-third experience difficulty interpreting health information independently [5]. Polypharmacy, common among the elderly, further increases the risk of adverse drug events, drug–drug interactions, and treatment burden, especially in patients with limited understanding of their therapeutic regimens [6]. Inadequate health literacy represents an important determinant of patient safety among geriatric populations.

Clinical pharmacists are uniquely positioned to address these challenges through comprehensive medication reviews, individualized counseling, medication reconciliation, and continuous patient education. Previous studies have reported associations between pharmacist-led interventions and improved medication adherence, therapeutic knowledge, and disease self-management in older adults [7]. A meta-analysis demonstrated that pharmacist counseling reduced medication-related problems by up to 45% and improved overall therapeutic outcomes in chronic disease populations [8]. Furthermore, pharmacist involvement in geriatric care has been associated with reduced hospital readmissions and enhanced patient satisfaction [9]. Despite these benefits, many elderly patients remain unaware of the full scope of clinical pharmacy services, limiting their utilization and potential impact [10].

Within Saudi Arabia, rapid demographic transitions have led to a marked increase in the older adult population, accompanied by a high prevalence of chronic illnesses such as hypertension, diabetes, cardiovascular disease, and arthritis [11]. These conditions require long-term pharmacotherapy and sustained patient engagement. However, recent evidence from the region suggests that health literacy among older adults remains suboptimal, and gaps persist in medication understanding and adherence [12]. Moreover, awareness of clinical pharmacist services, although expanding, remains limited among geriatric patients in many healthcare settings [13]. Addressing these gaps may support alignment with national health goals with patient-centered care, optimizing medication outcomes, and reducing preventable drug-related morbidity.

Given the expanding geriatric population in the Kingdom of Saudi Arabia and the critical role of pharmacists in enhancing medication safety, it is essential to evaluate the current level of health literacy, identify barriers related to medication management, and assess patients’ awareness of pharmacist-led services. This study aims to assess health literacy among elderly patients and explore their perceived needs, attitudes, and expectations toward clinical pharmacist involvement in their care. The findings are expected to inform practice enhancements, guide geriatric-focused pharmacy services, and support national strategies aimed at improving patient-centered care and chronic disease outcomes.

## 2. Methods

### 2.1. Study Design

This study employed a cross-sectional, questionnaire-based observational design to assess health literacy levels among geriatric patients and to evaluate their awareness and attitudes toward clinical pharmacist-led services. Cross-sectional approaches are widely used in geriatric health literacy assessments because they are appropriate for examining associations between health literacy and medication-related behaviors in outpatient settings [14,15]. This design is also appropriate for evaluating pharmacist service awareness in outpatient settings [16].

### 2.2. Study Setting

The study was conducted in outpatient clinics of a tertiary-care teaching hospital affiliated with King Khalid University, Saudi Arabia. Outpatient clinics represent key access points for elderly patients in chronic disease management and medication monitoring, making them ideal for assessing literacy and pharmacist-related needs [17].

### 2.3. Study Population and Eligibility

#### 2.3.1. Inclusion Criteria

Eligible participants were aged ≥60 years, consistent with WHO and Saudi definitions of the geriatric population [18], and were currently taking ≥2 chronic medications. Participants were required to be able to communicate verbally, be cognitively intact as determined by orientation questions assessing awareness of time, place, and person, and provide written or verbal informed consent.

#### 2.3.2. Exclusion Criteria

Participants were excluded if they had a diagnosed dementia or severe psychiatric illness impairing comprehension, were critically ill or clinically unstable, or declined participation. These criteria are consistent with international recommendations for literacy assessments in older adults [19].

### 2.4. Sampling Strategy and Sample Size

A convenience sampling technique was adopted, commonly used in outpatient literacy surveys where patient flow is variable but predictable [20]. A sample size of 200 participants was targeted, consistent with recommendations for single-center cross-sectional studies with categorical outcomes [21]. For the multivariable logistic regression analysis, sample adequacy was evaluated using the events-per-variable (EPV) criterion recommended by Peduzzi et al., which specifies a minimum of 10 outcome events per predictor variable included in the model [22]. With 6 predictor variables in the final model and 111 participants meeting the lower health literacy criterion, the EPV was 18.5, exceeding the recommended minimum threshold and confirming the adequacy of the sample for the planned multivariable analysis.

### 2.5. Data Collection Instruments

Three structured data collection tools were used. The Case Report Form (CRF) captured sociodemographic information, including age, sex, educational attainment, occupation, monthly income, and living arrangement, alongside clinical details such as chronic disease diagnoses, number of medications, frequency of hospital visits, and recent hospitalization history. Cognitive eligibility was also screened through the CRF using orientation questions assessing awareness of time, place, and person.

The Health Literacy Questionnaire assessed four functional domains: the ability to read and understand prescription labels; comprehension of instructions provided by physicians or nurses; confidence in independently completing medical forms; and understanding of written health information. The items were developed by the authors based on constructs from established health literacy frameworks, specifically the Health Literacy Survey—European Union (HLS-EU) model [23] and Baker’s conceptual framework [24], rather than directly reproducing any single validated instrument. As such, no formal permission for instrument reproduction was required; however, all conceptual sources have been explicitly cited. The tool should be interpreted as a purpose-built functional assessment aligned with, but not identical to, these validated frameworks.

The Pharmacist Awareness and Service Needs Questionnaire assessed participants’ awareness of clinical pharmacist roles, prior experience with medication counseling, preferred counseling format (face-to-face, leaflet, family-assisted, or telephone), and types of pharmacist support desired, including medication interaction checks, side-effect management, and test-report explanation.

These questionnaires were bilingual (English and Arabic) and interviewer-administered. The Arabic version was developed using a forward-backward translation procedure: the questionnaire was first translated into Arabic by a bilingual healthcare professional and then independently back-translated into English by a second bilingual translator blinded to the original version. Conceptual and semantic equivalence between versions was confirmed through research team consensus. The questionnaires were based on previously validated constructs [25].

The four-domain structure was developed in alignment with established health literacy frameworks, including the HLS-EU model [23], Baker’s conceptual framework of functional health literacy [24], and constructs assessed by validated instruments such as the Newest Vital Sign (NVS) and the Rapid Estimate of Adult Literacy in Medicine (REALM). Each domain reflects a functional literacy competency consistently identified across these validated instruments. While the tool was not independently subjected to psychometric validation in this study population, its conceptual grounding in established frameworks supports its use as a functional proxy measure of health literacy in geriatric outpatient settings.

### 2.6. Data Collection Procedure

Participants were approached in outpatient waiting areas using systematic screening. After confirming eligibility, informed consent was obtained following principles of autonomy and comprehension [26]. Interviews lasted approximately 5–10 min and were conducted in Arabic or English, depending on participant preference. The interviewer read each item aloud when needed to support individuals with visual impairments, common in older adults [27]. All questionnaires were completed on paper and later coded with unique participant IDs to ensure confidentiality. No personal identifiers (name, contact details) were collected.

### 2.7. Data Access Period

Participant recruitment and data collection were conducted between 20 November 2025 and 22 December 2025.

### 2.8. Operational Definition

Health literacy was operationally defined using a domain-based impairment model derived from Section A of the questionnaire. Four functional health-literacy domains were assessed: Domain 1 (Prescription label comprehension), based on the ability to read and understand prescription labels (Question 1); Domain 2 (Understanding and engagement with healthcare instructions), based on comprehension of healthcare providers’ instructions and the tendency to ask questions when information is unclear (Questions 2 and 3); Domain 3 (Form-filling confidence), based on self-reported confidence in completing medical forms independently (Question 4); and Domain 4 (Understanding written health information), based on comprehension of written health-related materials (Question 5). A domain was classified as impaired when responses indicated difficulty or inability in the corresponding item(s). Overall health-literacy impairment was quantified by the number of impaired domains (range: 0–4). For analytical purposes, participants were categorized into higher health literacy (0–2 domains impaired) and lower health literacy (3–4 domains impaired). The threshold of three or more impaired domains was selected to identify participants experiencing functional literacy difficulty across the majority of assessed domains, reflecting a clinically meaningful burden relevant to medication self-management. Each domain was assigned equal analytical weight, as all four represent distinct and equally relevant functional literacy competencies in the outpatient medication use context, and no empirical basis for differential domain weighting in geriatric populations was identified in the literature.

Medication mismanagement was defined as missing doses, incorrect dosing, inability to recall medication purpose, or failure to report experienced adverse drug reactions [6]. Although these behaviors are grouped under a single operational definition for conceptual clarity, each component was analyzed and reported separately in the results to allow independent interpretation of their associations with health-literacy impairment.

Pharmacist service awareness was defined as awareness that pharmacists provide services beyond dispensing, including medication counseling, interaction screening, and disease-specific education.

### 2.9. Ethical Considerations

This study was conducted in accordance with international research ethics guidelines and was approved by the Research Ethics Committee at King Khalid University (Approval No. KKU-41-2025-16, valid from 29 May 2025 to 28 May 2026). Participation in the study was entirely voluntary, and all individuals were informed about the purpose of the study, procedures, and their right to withdraw at any time without affecting their medical care. Informed consent was obtained prior to data collection.

To preserve confidentiality, no personal identifiers such as names or medical record numbers were collected. All questionnaires were coded and stored securely, and only aggregated data were used for analysis. The study ensured anonymity and minimized risk by employing a non-interventional, interview-based design. All procedures adhered to the ethical principles outlined in the Declaration of Helsinki [28], with special attention to respecting the autonomy and privacy of older adult participants.

### 2.10. Statistical Analysis

Data analysis was performed using IBM SPSS Statistics version 28.0 (IBM Corp., Armonk, NY, USA). Internal consistency of the health literacy items was assessed using Cronbach’s alpha prior to domain-based classification. Descriptive statistics were used to summarize demographic characteristics and key study variables, with categorical data presented as frequencies and percentages and continuous variables as means with standard deviations. To explore relationships between health literacy levels and participant characteristics, inferential analyses were conducted using the chi-square test or Fisher’s exact test when expected cell counts were small. Variables with a *p*-value < 0.20 in univariate analyses were considered candidate predictors for inclusion in the multivariable logistic regression model. This liberal threshold was intentionally applied to avoid premature exclusion of clinically relevant variables at the screening stage. In addition, variables considered a priori clinically relevant based on the established literature, including age, sex, educational attainment, income, comorbidity burden, and polypharmacy, were included in the model regardless of their univariate *p*-value. This combined data-driven and literature-informed variable selection approach is consistent with recommended practice for multivariable modeling in epidemiological research [22]. Prior to model building, multicollinearity among candidate predictors was assessed using the Variance Inflation Factor (VIF); all predictors retained in the final model had VIF values below 2.0, indicating no problematic collinearity. Model fit was evaluated using the Hosmer–Lemeshow goodness-of-fit test, and the proportion of variance explained was quantified using the Nagelkerke R^2^ statistic. A *p*-value < 0.05 was considered statistically significant throughout. This analytic approach is consistent with earlier geriatric health literacy research employing categorical outcome measures [4,12].

## 3. Results

### 3.1. Participant Characteristics (Table 1)

A total of 200 geriatric patients were included. The majority were aged ≥70 years (52.5%), and educational attainment was low, with 57.0% reporting illiterate or primary-level education. Full demographic and clinical characteristics are presented in Table 1.

**Table 1:** Baseline demographic and socioeconomic characteristics.

Characteristic	Category	*n* (%)
Age (years)	60–64	57 (28.5)
	65–69	38 (19.0)
	≥70	105 (52.5)
Sex	Male	102 (51.0)
	Female	98 (49.0)
Educational status	Illiterate	62 (31.0)
	Primary	52 (26.0)
	Secondary	64 (32.0)
	Graduate or above	22 (11.0)
Monthly income (SAR)	<5000	105 (52.5)
	5000–10,000	84 (42.0)
	>10,000	10 (5.0)
Living arrangement	Alone	3 (1.5)
	With spouse	59 (29.5)
	With family	138 (69.0)
Residence	Urban	116 (58.0)
	Semi-urban/Rural	84 (42.0)

HL: health literacy; SAR: Saudi Arabian Riyal.

### 3.2. Distribution of Health-Literacy Impairment (Table 2)

Health-literacy impairment was assessed using a domain-based approach across four functional domains. The five health literacy items demonstrated acceptable internal consistency (Cronbach’s alpha = 0.76). Overall, 55.5% of participants (*n* = 111) exhibited impairment in three or four domains, and 85.5% (*n* = 172) demonstrated impairment in two or more domains.

**Table 2:** Distribution of health-literacy impairment scores (N = 200).

Impaired Domains	*n* (%)
1 domain	28 (14.0)
2 domains	61 (30.5)
3 domains	74 (37.0)
4 domains	37 (18.5)
Total	200 (100.0)

Note: No participants were impairment-free (0 domains). All 200 participants demonstrated impairment in at least one domain.

### 3.3. Sociodemographic Predictors of Lower Health Literacy (Table 3)

Lower health literacy was significantly associated with older age, female sex, lower educational attainment, and lower monthly income. The strongest predictor was lower educational attainment (AOR: 3.21; 95% CI: 1.82–5.66), followed by lower income (AOR: 2.48; 95% CI: 1.41–4.38). Full results are presented in Table 3.

**Table 3:** Sociodemographic predictors of lower health literacy (3–4 domains impaired).

Variable	Category	Lower HL *n* (%)	Higher HL *n* (%)	*p*-Value	AOR (95% CI)
Age	≥70 years	79 (75.2)	26 (24.8)	0.002	2.34 (1.32–4.13)
	<70 years (Ref)	32 (33.7)	63 (66.3)	–	1.00 (Ref)
Sex	Female	63 (64.3)	35 (35.7)	0.031	1.67 (1.05–2.66)
	Male (Ref)	48 (47.1)	54 (52.9)	–	1.00 (Ref)
Education	Illiterate/Primary	86 (75.4)	28 (24.6)	<0.001	3.21 (1.82–5.66)
	≥Secondary (Ref)	25 (29.8)	59 (70.2)	–	1.00 (Ref)
Income (SAR)	<5000	77 (73.3)	28 (26.7)	<0.001	2.48 (1.41–4.38)
	≥5000 (Ref)	34 (36.6)	59 (63.4)	–	1.00 (Ref)

Lower HL: lower health literacy (3–4 domains impaired); Higher HL: higher health literacy (0–2 domains impaired); AOR: adjusted odds ratio; CI: confidence interval; Ref: reference category; SAR: Saudi Arabian Riyal.

### 3.4. Clinical Burden and Lower Health Literacy (Table 4)

Among clinical factors, polypharmacy was strongest independent predictor of lower health literacy (AOR: 3.46; 95% CI: 1.93–6.21), followed by multimorbidity (AOR: 2.91; 95% CI: 1.66–5.10). Full results are presented in Table 4.

**Table 4:** Clinical burden and lower health literacy.

Variable	Category	Lower HL *n* (%)	Higher HL *n* (%)	*p*-Value	AOR (95% CI)
Comorbidities	≥3	83 (72.8)	31 (27.2)	<0.001	2.91 (1.66–5.10)
	1–2 (Ref)	28 (32.9)	57 (67.1)	–	1.00 (Ref)
Polypharmacy	≥5 drugs	92 (78.0)	26 (22.0)	<0.001	3.46 (1.93–6.21)
	2–4 drugs (Ref)	19 (23.2)	63 (76.8)	–	1.00 (Ref)
Hospitalization (past year)	Yes	55 (70.5)	23 (29.5)	0.004	1.98 (1.23–3.60)
	No (Ref)	56 (45.9)	66 (54.1)	–	1.00 (Ref)

Lower HL: lower health literacy (3–4 domains impaired); Higher HL: higher health literacy (0–2 domains impaired); AOR: adjusted odds ratio; CI: confidence interval; Ref: reference category.

### 3.5. Health-Literacy Level and Medication Self-Management (Table 5)

Participants with lower health literacy reported significantly higher rates of missed or incorrect dosing and unreported adverse drug reactions and were less likely to use medication management aids. Full results are presented in Table 5.

**Table 5:** Health-literacy level and medication self-management.

Outcome	Lower HL *n* (%)	Higher HL *n* (%)	*p*-Value	AOR (95% CI)
Missed or incorrect dose—Yes	69 (62.2)	26 (29.2)	<0.001	2.71 (1.61–4.58)
Missed or incorrect dose—No	42 (37.8)	63 (70.8)	–	–
Unreported ADRs—Yes	47 (42.3)	15 (16.9)	0.001	2.26 (1.29–3.97)
Unreported ADRs—No	64 (57.7)	74 (83.1)	–	–
Uses medication aid—Yes	21 (18.9)	42 (47.2)	<0.001	0.39 (0.22–0.69)
Uses medication aid—No	90 (81.1)	47 (52.8)	–	–

Lower HL: lower health literacy (3–4 domains impaired); Higher HL: higher health literacy (0–2 domains impaired); AOR: adjusted odds ratio; CI: confidence interval; ADRs: adverse drug reactions. Complementary ‘No’ rows provided for complete distribution. AOR reported for ‘Yes’ outcomes only.

### 3.6. Awareness of Pharmacist Services by Health-Literacy Level (Table 6)

Lower health literacy was strongly associated with unawareness of pharmacist roles (AOR: 3.28; 95% CI: 1.89–5.69) and lower prior exposure to pharmacist counseling (AOR: 2.74; 95% CI: 1.55–4.85). Full results are presented in Table 6.

**Table 6:** Awareness of pharmacist services by health literacy level.

Variable	Lower HL *n* (%)	Higher HL *n* (%)	*p*-Value	AOR (95% CI)
Unaware of pharmacist’s role—Yes	89 (80.2)	26 (29.2)	<0.001	3.28 (1.89–5.69)
Unaware of pharmacist’s role—No	22 (19.8)	63 (70.8)	–	–
Never received counseling—Yes	96 (86.5)	44 (49.4)	<0.001	2.74 (1.55–4.85)
Never received counseling—No	15 (13.5)	45 (50.6)	–	–

Lower HL: lower health literacy (3–4 domains impaired); Higher HL: higher health literacy (0–2 domains impaired); AOR: adjusted odds ratio; CI: confidence interval. Complementary rows provided for complete distribution.

### 3.7. Willingness to Receive Pharmacist Counseling (Table 7)

Willingness to receive pharmacist counseling was significantly higher among participants with higher health literacy and those aware of pharmacist services. Full results are presented in Table 7.

**Table 7:** Willingness to receive pharmacist counseling by health literacy and awareness.

Predictor	Category	Willing *n* (%)	Not Willing/Uncertain *n* (%)	*p*-Value	AOR (95% CI)
Health literacy	Higher HL (Ref)	76 (85.4)	13 (14.6)	–	1.00 (Ref)
	Lower HL	57 (51.4)	54 (48.6)	<0.001	0.41 (0.23–0.73)
Pharmacist awareness	Aware (Ref)	71 (84.5)	13 (15.5)	–	1.00 (Ref)
	Not aware	62 (53.9)	53 (46.1)	<0.001	3.88 (2.01–7.51)

Higher HL: higher health literacy (0–2 domains impaired); Lower HL: lower health literacy (3–4 domains impaired); AOR: adjusted odds ratio; CI: confidence interval; Ref: reference category.

### 3.8. Multivariable Regression Analysis for Lower Health Literacy (Table 8)

In the multivariable logistic regression model, several factors remained independently associated with lower health literacy after adjustment for relevant covariates. Participants with illiterate or primary-level education had significantly higher odds of lower health literacy (AOR: 3.21; 95% CI: 1.82–5.66; *p* < 0.001). Polypharmacy (≥5 medications) was also a strong independent predictor (AOR: 3.46; 95% CI: 1.93–6.21; *p* < 0.001), as was the presence of three or more comorbidities (AOR: 2.91; 95% CI: 1.66–5.10; *p* < 0.001). Lower monthly income (<5000 SAR) remained significantly associated with lower health literacy (AOR: 2.48; 95% CI: 1.41–4.38; *p* = 0.002). Female sex was also independently associated with lower health literacy (AOR: 1.67; 95% CI: 1.05–2.66; *p* = 0.031). Additionally, lack of awareness of clinical pharmacist services was a significant predictor of lower health literacy (AOR: 3.28; 95% CI: 1.89–5.69; *p* < 0.001). The model demonstrated good fit (Hosmer–Lemeshow *p* = 0.59) and explained a substantial proportion of variance (Nagelkerke R^2^ = 0.46).

**Table 8:** Multivariable logistic regression model for lower health literacy (3–4 domains impaired).

Predictor	Adjusted OR	95% CI	*p*-Value
Illiterate/Primary education	3.21	1.82–5.66	<0.001
Polypharmacy (≥5 drugs)	3.46	1.93–6.21	<0.001
≥3 comorbidities	2.91	1.66–5.10	<0.001
Lower income (<5000 SAR)	2.48	1.41–4.38	0.002
Female sex	1.67	1.05–2.66	0.031
Lack of pharmacist awareness	3.28	1.89–5.69	<0.001

AOR: adjusted odds ratio; CI: confidence interval; SAR: Saudi Arabian Riyal. Model adjusted for age, sex, educational attainment, income, comorbidity burden, and medication count. Model fit: Hosmer–Lemeshow *p* = 0.59; Nagelkerke R^2^ = 0.46.

## 4. Discussion

This study assessed functional health literacy among geriatric outpatients and examined its relationship with medication self-management and perceptions of pharmacist-led services. Using a four-domain impairment model, a high proportion of participants demonstrated impairment across multiple domains, and lower health literacy (3–4 domains) was independently associated with older age, lower education, lower income, female sex, multimorbidity, and polypharmacy. In addition, low health literacy was associated with less favorable medication self-management behaviors and lower awareness of pharmacist roles, while willingness to receive pharmacist counseling was higher among those with higher health literacy and greater awareness.

### 4.1. Lower Health Literacy and Sociodemographic Vulnerability

The observed clustering of lower health literacy among older participants and those with lower educational attainment and lower income is consistent with recent findings from Web of Science-indexed studies showing that functional and medication-related health literacy are socially patterned and disproportionately affect vulnerable older populations [29,30]. Similar associations have been reported in recent cross-sectional studies examining determinants of medication literacy among older adults, where education and socioeconomic status remained strong predictors after multivariable adjustment [31]. These findings reinforce the relevance of incorporating literacy-sensitive considerations into geriatric outpatient care and medication management strategies.

The independent association between female sex and lower health literacy observed in this study is particularly meaningful in the Saudi Arabian context. The current generation of older women in Saudi Arabia (aged ≥60 years) came of age during a period when female access to formal education was significantly restricted. Public education for girls was only formally established in Saudi Arabia in 1960, meaning that women in this age group had limited or no access to schooling during their formative years. Consequently, illiteracy rates among older Saudi women remain substantially higher than among their male counterparts, with national surveys consistently reporting lower educational attainment among elderly females [32]. Beyond formal education, this generation of women may have had limited exposure to healthcare navigation, reduced autonomy in medical decision-making, and lower engagement with written health materials—all of which are likely to compound functional health-literacy impairment. These sociohistorical factors provide important context for interpreting the observed sex-based disparity and highlight the need for gender-sensitive, literacy-adapted approaches in geriatric pharmacist-led care.

### 4.2. Clinical Complexity: Polypharmacy, Multimorbidity, and Health Literacy

In the present analysis, polypharmacy and higher comorbidity burden emerged as strong independent predictors of lower health literacy. Recent systematic reviews and observational studies indexed in Web of Science have demonstrated that complex medication regimens and multimorbidity are closely linked to poorer medication understanding, reduced adherence, and increased medication-related risk, with health literacy acting as an important contributing factor [29,33]. Related work among older adults has further shown that limited health literacy is associated with higher medication burden and suboptimal therapeutic outcomes, supporting the associations observed in this study [32]. Although causality cannot be inferred from the current design, the consistency of these findings across diverse settings strengthens their external relevance.

### 4.3. Medication Self-Management Outcomes and Domain-Based Impairment

Participants with lower health literacy were more likely to report missed or incorrect dosing and unreported adverse drug reactions, while the use of medication management aids was lower in this group. These findings align with recent evidence indicating that limited health literacy is associated with poorer medication self-management behaviors among older adults [30,34]. Recent meta-analyses and systematic reviews have further reported that medication-focused interventions, including pharmacist-led medication reviews and counseling, are associated with improvements in medication-related processes and safety outcomes in older populations [33,35]. While the present study did not evaluate intervention effects, the observed associations highlight potential targets for future pharmacist-led strategies.

### 4.4. Awareness and Willingness to Engage with Pharmacist-Led Services

Lower awareness of pharmacist roles and lower exposure to pharmacist counseling were observed among participants with lower health literacy. This pattern is consistent with recent studies reporting gaps in patient understanding of clinical pharmacy services and variability in service utilization, even in healthcare systems where pharmacist roles are expanding [36,37]. In contrast, willingness to receive pharmacist counseling was significantly higher among participants who were aware of pharmacist-provided services, suggesting that awareness may play a key role in facilitating engagement. Similar findings have been reported in recent studies examining the relationship between health literacy, provider awareness, and patient engagement in medication management [31,36].

### 4.5. Strengths and Limitations

This study applies a pragmatic domain-based model to assess functional health literacy relevant to outpatient medication use and pharmacist counseling. The use of multivariable analysis allowed the identification of independent predictors consistent with contemporary literature [29,30,31,32,33,34]. However, several limitations should be acknowledged. The cross-sectional design precludes causal inference. Participants were recruited using convenience sampling from a single tertiary-care outpatient center, which limits the generalizability of findings to broader geriatric populations in Saudi Arabia or other settings. Although recruitment was conducted across multiple outpatient specialty clinics and varied clinic days throughout the data collection period to introduce patient diversity, these measures do not fully overcome the inherent constraints of single-site convenience sampling. Additionally, the inclusion of participants taking as few as two chronic medications introduced heterogeneity within the non-polypharmacy comparison group, which may have influenced the magnitude of the observed polypharmacy association. Furthermore, the study was conducted exclusively in a tertiary-care teaching hospital outpatient setting. Geriatric patients accessing community pharmacies, primary care clinics, or long-term care and nursing home facilities may present with different health literacy profiles, medication management challenges, and levels of pharmacist service awareness. The applicability of these findings to such settings should therefore be interpreted with caution. Medication-related behaviors and adverse drug reactions were assessed through self-report, introducing two important potential biases. First, recall bias may have affected the accuracy of participants’ accounts of past medication-taking behaviors, missed or incorrect doses, and experienced adverse drug reactions, particularly given the absence of a defined reference period and the known cognitive variability in older adult populations. Second, social desirability bias may have led participants to report more favorable medication behaviors than actually occurred, as the interviewer-administered format—although necessary to accommodate participants with visual impairment or low literacy—may have encouraged responses perceived as socially acceptable to healthcare staff. These biases may have resulted in underestimation of medication mismanagement and adverse drug reaction rates, and findings should be interpreted with this in mind. In addition, while the domain-based approach offers clinical practicality, its four-domain structure was developed in alignment with established health literacy frameworks, including the Health Literacy Survey—European Union (HLS-EU) model [22], Baker’s conceptual framework of functional health literacy [23], and constructs assessed by validated instruments such as the Newest Vital Sign (NVS) and the Rapid Estimate of Adult Literacy in Medicine (REALM). However, it was not independently psychometrically validated in this study population and should therefore be interpreted as a functional proxy measure rather than a substitute for comprehensive, validated health literacy instruments.

### 4.6. Implications and Future Directions

The findings indicate that older adults with greater clinical complexity and social vulnerability are more likely to exhibit lower health literacy, poorer medication self-management, and lower awareness of pharmacist services. Future research could evaluate targeted pharmacist-led interventions incorporating literacy-sensitive communication strategies, simplified educational materials, and medication management aids. Longitudinal and interventional studies are also warranted to assess whether improving health literacy and pharmacist engagement leads to measurable improvements in medication safety and health outcomes among geriatric populations. Future research should also extend beyond tertiary-care outpatient settings to examine health literacy and pharmacist service awareness among geriatric patients in community pharmacy, primary care, and long-term care settings, where patient profiles and care dynamics may differ substantially.

## 5. Conclusions

Health-literacy impairment was common among geriatric outpatients and frequently involved multiple functional domains. Lower health literacy was independently associated with older age, lower educational attainment, lower income, female sex, multimorbidity, and polypharmacy. Participants with lower health literacy demonstrated poorer medication self-management, including higher rates of missed dosing and unreported adverse drug reactions, and lower use of medication aids. Awareness of and prior exposure to clinical pharmacist services were significantly lower among those with lower health literacy, while willingness to engage with pharmacist counseling was higher among aware participants. These findings underscore the importance of functional health literacy in geriatric medication management and support the development of literacy-sensitive pharmacist-led interventions.

## Data Availability

The data supporting the findings of this study are available at https://doi.org/10.5281/zenodo.19349586.
